# Axial Compressive Strength Models of Eccentrically-Loaded Rectangular Reinforced Concrete Columns Confined with FRP

**DOI:** 10.3390/ma14133498

**Published:** 2021-06-23

**Authors:** Haytham F. Isleem, Muhammad Abid, Wesam Salah Alaloul, Muhammad Kamal Shah, Shayan Zeb, Muhammad Ali Musarat, Muhammad Faisal Javed, Fahid Aslam, Hisham Alabduljabbar

**Affiliations:** 1 Department of Civil Engineering, Tsinghua University, Beijing 100084, China; isleemhaytham88@gmail.com; 2 College of Aerospace and Civil Engineering, Harbin Engineering University, Harbin 150001, China; mrkamalshah@outlook.com (M.K.S.); shayanzeb62@outlook.com (S.Z.); 3 Department of Civil and Environmental Engineering, Universiti Teknologi PETRONAS, Seri Iskandar 32610, Perak, Malaysia; muhammad_19000316@utp.edu.my; 4 Department of Civil Engineering, Abbottabad Campus, COMSATS University Islamabad, Abbottabad 22060, Pakistan; arbabfaisal@cuiatd.edu.pk; 5 Department of Civil Engineering, College of Engineering in Al-Kharj, Prince Sattam bin Abdulaziz University, Al-Kharj 11942, Saudi Arabia; f.aslam@psau.edu.sa (F.A.); h.alabduljabbar@psau.edu.sa (H.A.)

**Keywords:** FRP-confined concrete, FRP rupture strain, rectangular sections, strength model, eccentric axial compression, large-scale RC columns

## Abstract

The majority of experimental and analytical studies on fiber-reinforced polymer (FRP) confined concrete has largely concentrated on plain (unreinforced) small-scale concrete columns, on which the efficiency of strengthening is much higher compared with large-scale columns. Although reinforced concrete (RC) columns subjected to combined axial compression and flexural loads (i.e., eccentric compression) are the most common structural elements used in practice, research on eccentrically-loaded FRP-confined rectangular RC columns has been much more limited. More specifically, the limited research has generally been concerned with small-scale RC columns, and hence, the proposed eccentric-loading stress-strain models were mainly based on the existing concentric-loading models of FRP-confined concrete columns of small scale. In the light of such demand to date, this paper is aimed at developing a mathematical model to better predict the strength of FRP-confined rectangular RC columns. The strain distribution of FRP around the circumference of the rectangular sections was investigated to propose equations for the actual rupture strain of FRP wrapped in the horizontal and vertical directions. The model was accomplished using 230 results of 155 tested specimens compiled from 19 studies available in the technical literature. The test database covers an unconfined concrete strength ranging between 9.9 and 73.1 MPa, and section’s dimension ranging from 100–300 mm and 125–435 mm for the short and long sides, respectively. Other test parameters, such as aspect ratio, corner radius, internal hoop steel reinforcement, FRP wrapping layout, and number of FRP wraps were all considered in the model. The performance of the model shows a very good correlation with the test results.

## 1. Introduction

The application of fiber-reinforced polymers (FRPs) has been abundantly used to retrofit concrete columns in existing buildings and bridges. The importance of this subject has been confirmed by numerous researchers and their studies reported that the axial compression strength and ductility of concrete columns under pure concentric loading can be substantially increased using the FRP wrapping system (e.g., [1,2,3,4,5]).

Due to their high strength/stiffness-to-weight ratios, fiber-reinforced composites have become increasingly popular in aerospace, aviation as well as in civil engineering structures [6,7,8]. In real buildings, concrete columns are subject to eccentric loads, i.e., flexural loadings and combined axial compression resulted due to construction errors and accidental load eccentricities resulted from vehicular loads or earthquake loads. With regards to design rules, when concrete elements are subjected to eccentric loads, it is crucial to assess the increase in loading eccentricity, as tensile stresses can develop in a part of the cross section, necessitating additional reinforcement. If the effects of increasing eccentricity during their service is not considered, the load carrying capacity of the designed elements are not sufficient [9,10,11,12,13,14]. Many studies have been carried out to investigate the compressive axial behavior of FRP-confined concrete columns when subjected to eccentric loads. A brief review of the relevant literature is provided in the following.

FRP-confined circular unreinforced and reinforced concrete columns were tested by Hadi et al. [15,16] and Li and Hadi [17] to investigate the column behavior under varying eccentricities. The results indicated that external confinement of columns with FRP composites can significantly enhance the strength, when subjected to eccentric loads. Additionally, the studies demonstrated that varying eccentric loads reduced the effectiveness of FRP confinement. The study of Hadi and Widiarsa [18] yielded insights into the performance of eccentrically-loaded CFRP-confined square high-strength concrete columns with a section width of 200 mm and a height of 800 mm. The study investigated the influence of a varying number of CFRP layers, varying eccentricity ratio (i.e., 0.125–0.25 *h*), and the use of longitudinal CFRP straps. The results clearly showed that confinement with CFRP enhanced the columns load carrying capacity under eccentric loads. In existing tests, the use of longitudinal CFRP straps, in case of large eccentricity, significantly enhanced the overall performance of the columns.

El Maaddawy et al. [19] and Lie et al. [20] investigated the influence of the cross-sectional shape on the behavior of RC columns strengthened with CFRP and subjected to different loading cases. The tests by El Maaddawy et al. [19] included parameters, such as cross-sectional shape (i.e., circular, square, and rectangular), different aspect ratios (i.e., *h/b* = 1, 1.25, 1.45), and the loading condition (i.e., concentric and eccentric loads). The studies revealed that the shape of the cross sections influenced the overall column performance. The gain in the load carrying capacity of CFRP-confined columns with rectangular sections decreases with increasing the level of eccentricity and the aspect ratio of cross sections (e.g., El Maaddawy et al. [19]). The influence of the cross-sectional shape on CFRP tensile strains was also investigated, in which the CFRP hoop tensile strains of the eccentrically loaded column sections were significantly lower than those of the concentrically loaded columns. Additional reduction in the CFRP tensile strains was also investigated due to a second-order effect experienced by the increase of eccentricity.

Hassan et al. [21] tested 200 mm × 200 mm × 1050 mm high-strength concrete short columns strengthened by Glass FRP wraps to investigate the effect of arrangement and amount of FRP wraps. The tested parameters included the number of layers of FRP wraps in the hoop direction (i.e., partial or full wrapping), eccentric loading condition (i.e., small and large eccentricities), and the number of FRP layers along the longitudinal tension side of the columns. The study revealed that the confinement using GFRP-strengthening contributed to the enhancement of the load carrying capacity. Furthermore, the use of longitudinal GFRP layers overlaid by FRP wraps partially applied in the hoop direction is a successful method to enhance both the load and the flexural capacities.

Yang et al. [22] studied the structural performance of eccentrically loaded columns through tests on CFRP-strengthened high strength concrete columns with a rectangular cross section of 150 mm × 200 mm and a height of 1200 mm. The influence of eccentric loading ratio (i.e., *e/h* = 0.25–0.5), wrapping scheme (i.e., partial and full wrapping), the number of CFRP layers (i.e., 1, 2, 3, 4), and pre-damaged condition was investigated. The test results generally indicated an increase in the ultimate load of the CFRP-wrapped columns. Regarding the hoop strain of CFRP strips at the mid-height of the column, similar findings to those reported by El Maaddawy et al. [19] have also been reached by Yang et al. [22], which reveal a decrease in their values as the eccentricity increased.

Among the existing studies, there are only very few studies available on concentrically-loaded FRP-confined RC columns that address the strength enhancement caused by the confinement from using external FRP confinement and internal steel confinement (e.g., [10,11,12,13,14,23,24,25,26]). Of these studies, a series of 68 axial compressive tests on FRP-wrapped reinforced concrete in 150 mm × 300 mm rectangular-sectioned and 250 mm × 250 mm square and circular-sectioned columns of 500 mm in height were conducted by Ilki et al. [24]. Wang et al. [10,11] recently conducted research to develop a stress-strain model for large-scale RC columns with square cross sections enclosed in CFRP wraps. The results indicated that longitudinal steel reinforcing bars and the internal hoops contribute to the improvement of the axial strength of the specimens. In these studies, the lateral pressure was derived from the confinement from the FRP wraps and the internal steel hoops. For eccentrically loaded RC columns, Jaturapitakkul et al. [27] investigated the effect of internal steel confinement on the axial compression response of columns under eccentric loads. Reinforced concrete columns with and without internal confinement were tested for different eccentric ratios. The columns with steel hoops of smaller spacing have shown to be more effective in enhancing the ultimate load as compared with the ones with larger hoop spacing.

Most of the existing literature on eccentrically-loaded FRP-confined columns are experimental studies. Based on an experimental program consisted of tests on FRP-confined square and rectangular RC columns subjected to concentric and eccentric compression loading, Song et al. [28] provided an analytical formula for the maximum compressive load with respect to unconfined columns based on a regression analysis of parametric study results. In their study, FRP-confined square RC columns were tested to verify the proposed model. The effects of eccentricity ratio, FRP-confinement ratio, and unconfined concrete strength on the enhancement provided by the FRP strengthening ratio were identified. The mathematical form of the model is provided in Equation (1).

(1) $$\begin{array}{l} \frac{p_{uc}}{p_{u}}=1+1.66e^{-0.06f_{c}^{\prime }}\times \left(181.8\rho_{f}+0.66\right)\\ \times \left\{ \begin{array}{l} 1.79-2.84\frac{e}{h}\;if\;\rho_{f}=0.0267\\ 1.04-1.48\frac{e}{h}\;if\;\rho_{f}=0.016\\ 0.193\;if\;\rho_{f}=0.00267 \end{array}\right.\;(e/h\leq 0.5) \end{array}$$

The amount of FRP used was quantified by a confinement ratio, *ρ_f_*, which was calculated on the basis of the volumetric ratio of FRP wraps: *ρ_f_* = 4*n_f_t_f_/D*, where *n* and *t_f_* (mm) are the number of layers and the thickness of the FRP fabric, respectively, and *D* (mm) is the equivalent diameter of square and rectangular column sections, taken as $D=\sqrt{b^{2}+h^{2}}$. Based on their material test properties, the formula consisted of a volumetric FRP ratio without considering the mechanical properties of the FRP sheets. However, an adjustment factor to account for the differences in mechanical properties to that used in their study was considered as *t_f,a_* = *t_f_* × *f_frp_*/*f_frp,r_*, where in analysis the *f_frp,r_* is considered as the reference tensile strength of FRP sheets and taken to be 3500 MPa.

**Figure 1:**
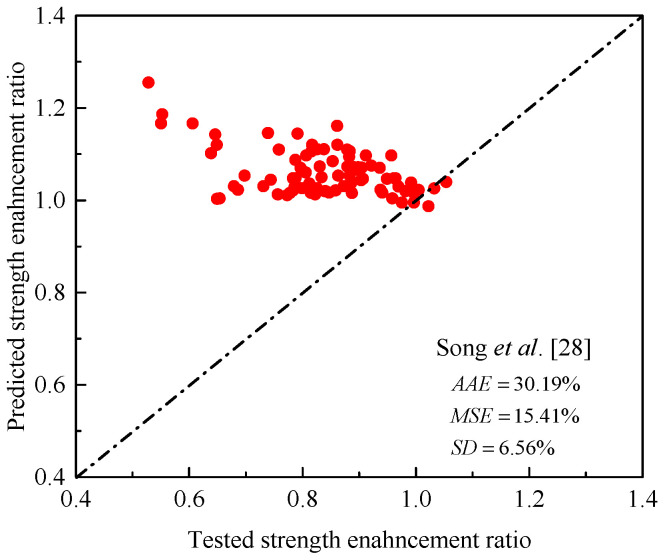
Comparison between tested and maximum load ratios by the model of Song et al. [28] for FRP-confined RC columns.

The performance of the Song et al. [28] model was checked using three statistical indicators determined using Equations (2)–(4), namely (1) average absolute error (*AAE*), (2) mean square error (*MSE*), and (3) standard deviation (*SD*), where *n* = total number of the test data. Figure 1 shows a clear comparison between the experimental and predicted data of a total of 84 tested specimens compiled from the literature. It is clearly shown that the use of their model leads to significant errors in predicting the maximum strength of the test columns.

(2) $$AAE=\frac{\sum_{i=1}^{n}\left|\frac{mod_{i}-exp_{i}}{exp_{i}}\right|}{n}$$

(3) $$MSE=\frac{\sum_{i=1}^{n}{\left(\frac{mod_{i}-exp_{i}}{exp_{i}}\right)}^{2}}{n}$$

(4) $$SD=\sqrt{\frac{\sum_{i=1}^{n}{\left(\frac{mod_{i}}{exp_{i}}-\frac{mod_{avg}}{exp_{avg}}\right)}^{2}}{n-1}}$$

Although there are only limited analytical investigations on FRP-confined concrete columns under eccentric loading, these models have not considered the effects of several important parameters as summarized in this Introduction. A summary of the models is provided in Table 1. Therefore, this paper provides a new model for larger-size FRP-confined rectangular RC columns. The proposed model is based on tests compiled from the available literature and also has a similar approach to that reported by Song et al. [28] but with several modifications. The confining pressure at the corners of the FRP-confined sections is first modeled using the FRP tensile properties and the details of corner radii and cross-sectional dimensions. A model is finally proposed to estimate the strength of confined columns, which was evaluated by the same data used in the model development. A comparison made between the analytical and test data demonstrated the accuracy and validity of the performed analysis.

## 2. Experimental Program

### 2.1. Test Database

To develop a model and to verify its accuracy, a more comprehensive database of 230 results from eccentrically-loaded FRP-confined concrete specimens with varying geometric and material properties was assembled from the existing literature [18,19,21,22,27,28,29,32,33,34,35,36,37,38,39,40,41,42,43]. To examine the effect of unconfined concrete strength on the effectiveness of FRP confinement as conducted in concentrically loaded models (e.g., [44,45,46,47]), the database contains data on unconfined concrete strengths ranging from 9.9 MPa to 73.1 MPa. Various types of FRP were tested in the previously noted experiments, namely glass FRP (GFRP), aramid FRP (AFRP) and carbon FRP (CFRP). All specimens were reinforced with longitudinal and hoop steel bars with the exception of the specimens described by Darby et al. [33] which only included longitudinal steel bars. All specimens were also strengthened using FRP wraps except the specimens of Jaturapitakkul et al. [27] which were only confined by internal steel hoops. The analysis considered a variety of different FRP confinement levels. The specimen dimensions range from 100 mm to 300 mm and 125 mm to 435 mm for the shorter and longer sides, respectively. The aspect ratio of the cross sections ranged from 1 to 1.5 and the total height ranged from 500 mm to 2700 mm. For most specimens, the physical properties of FRP were provided by manufacturers with the exception of those determined from flat coupon tensile tests by Darby et al. [33], Hadi et al. [18], Song et al. [28], and Yang et al. [22]. Table 2 displays a summary of the test database and the mechanical properties of the steel reinforcement and the FRP material are presented in Table 3. For an accurate analysis, the following points in data manipulation are also considered.

In the studies reported by Xian et al. [39] and Hassan et al. [21], rounded rectangular sections were confined with FRP wraps. Information regarding the corner radius dimension was not provided. For the purpose of the current analytical modeling, a minimum corner radius of the rectangular sections is, therefore, assumed as recommended in the International Codes (*r_c_* = 13 mm) (ACI 440.2R-08 [47]).

In the studies reported by El Maaddawy [29], El Maaddawy et al. [19], Allawi et al. [35], and Shaikh and Alishahi [34], different results for the same SikaWrap-230C type were provided. For an accurate modeling, this paper therefore adopts one unique result that was found by El Maaddawy [29].

Columns with unconfined concrete strength derived from 150 mm × 200 mm cylinders and 150 mm × 150 mm cubes were converted to standard cylinder using the following expressions proposed in this paper. Equation (5) is based on the results provided by Cheng et al. [48], whereas Equation (6) is based on the results published by Reddy et al. [49].

(5) $$f_{c}^{\prime }=0.503+0.572{\left(f_{cd}^{\prime }\right)}^{1.107}$$

(6) $$f_{c}^{\prime }=\left(0.0028{\left(f_{cu}^{\prime }\right)}^{1.1803}-0.0052f_{cu}^{\prime }+0.7980\right)\times f_{cu}^{\prime }$$

where $f_{c}^{\prime }$ (MPa) is the compressive strength of unconfined concrete standard cylinders; $f_{cd}^{\prime }$ and $f_{cu}^{\prime }$ (MPa) are the unconfined compressive strength of small-sized cylinders and cubes, respectively.

### 2.2. Failure Modes and Distribution of FRP Strains

Under concentric loading, tests on FRP-confined concrete columns revealed that the specimens failed abruptly due to tensile rupture of the CFRP wrap within the mid-height region, with no failure occurred near the columns ends due to end strengthening (e.g., [10,12]). Rupture originated at or near the corners of the sections and the axial strength of the columns was suddenly lost when the extent of FRP rupture was considerable (e.g., [50,51,52]).

Experiments on eccentrically-loaded FRP-confined columns demonstrate that specimen failure is primarily determined by the magnitude of the eccentricity. Failure patterns of columns tested under small eccentricity ratios (i.e., *e/h* = 0.175, 0.250, 0.325) were almost similar, where crushing of concrete leads to sudden and explosive rupture of the CFRP wraps at the compression size (e.g., Al-Nimry and Neqresh [32]). Local buckling of the longitudinal steel bars was also observed underneath the ruptured FRP jacket in the compression zone. Similar results were also reported by Darby et al. [33] for columns with *e/h* = 0.10 and 0.327. However, FRP-confined columns under relatively larger eccentricities failed by yielding of tension steel that followed by the rupture of CFRP wraps at the compression side (e.g., El Maaddawy et al. [29]). This demonstrates that at an *e/h* value of 0.43 and 0.57, respectively, the mode of failure changed from compression and balanced failure modes to a tension mode of failure. Similarly, for specimens with eccentricity ratios of 0.72 and 0.753, failure of the FRP-confined columns was attributed by yielding of the longitudinal steel bars on the tension side (e.g., Darby et al. [33]).

The assumption that stresses of an effectively FRP-confined rectangular concrete core are equal, as for a circular column under concentric load, is not valid. The distribution of the FRP hoop strain around the perimeter of the rectangular cross sections is not uniform. Research observations indicated that the rupture of FRP typically originated at or near the cross section’s corners in the compression zone. Furthermore, when the FRP-confined columns are subjected to eccentric loads, the confining forces are not equal at each corner (e.g., Darby et al. [33]). As a result, the mechanism of the FRP-confined concrete in eccentrically loaded rectangular sections should concentrate on the FRP hoop strain at the corners as for concentrically-loaded FRP-confined columns by Pham and Hadi [51], Wang et al. [9,10,11] and Isleem et al. [12,13,14].

## 3. Analytical Modeling

### 3.1. Rupture Strain of FRP in Rectangular Cross Sections

The FRP rupture strain for rectangular-sectioned columns with small corner radii, has been shown to be significantly lower than the tensile failure strain obtained from pure tensile tests (e.g., Campione and Miraglia [53], Barrington et al. [54]). According to the literature, Wang and Wu [55] tested FRP-confined concrete specimens to investigate the effect of corner radius on the rupture strain of FRP under pure axial compression. Their investigation led them to conclude the rupture strain of FRP generally increases as the radius of the corners increases. It is, therefore, assumed in the current model that the FRP rupture strain is dependent on the side length and on the ratio of the corner radius, which could be 2*r_c_*/*h* or 2*r_c_*/*b* as used for FRP-confined rectangular sections tested by Isleem et al. [12,13,14].

Another important parameter that influences the rupture strain of FRP is the size of the cross section. In non-circular sections (i.e., rectangular), it was found by Yan [56] that the ratios of CFRP rupture strain to the actual tensile strain of the CFRP were 40 and 60% for the larger and smaller dimensioned rectangular columns, respectively. The average CFRP rupture strains for large-size (*b* > 300 mm) and medium-size (150 mm < *b* < 300 mm) square sectioned columns were 40 and 60% of the ultimate tensile strain, respectively (Wang et al. [10,11]).

Furthermore, research by Pham and Hadi [51] has shown that the actual rupture strain of the FRP depends on the confinement stiffness ratio denoted as *R_s_*, which was defined by Teng et al. [57] as

(7) $$R_{s}=\frac{2n_{f}t_{f}E_{f}}{\left(\frac{f_{c}^{\prime }}{\varepsilon_{co}}\right)D}$$

where $f_{c}^{\prime }$ (MPa) and *ε_co_* (mm/mm) are the compressive strength of unconfined concrete cylinders and its corresponding strain; *n_f_* = number of FRP layers; *E_f_* (MPa) = tensile elasticity modulus of FRP composites (MPa); *t_f_* (mm) = nominal thickness of FRP wraps; *D* (mm) is the diameter of a circular section. The unconfined concrete strain, *ε_co_*, was estimated using Equation (8) provided by Tasdemir et al. [58].

(8) $$\varepsilon_{co}=(-0.067f_{c}^{\prime 2}+29.9f_{c}^{\prime }+1053)\times 10^{-6}$$

In an analytical investigation of Pham and Hadi [51], Equation (7) is modified by replacing *D*/2 with *r_c_*, which is the corner radius of a rectangular cross section (mm). As the current paper deals with partially and fully wrapped RC columns, as well as columns additionally reinforced by longitudinal FRP sheets, Equations (9) and (10) are provided, respectively.

(9) $$R_{sh}=\left\{ \begin{array}{l} \frac{n_{f}t_{f}E_{f}}{\left(\frac{f_{c}^{\prime }}{\varepsilon_{co}}\right)r_{c}}\\ \frac{n_{f}t_{f}w_{f}E_{f}}{\left(\frac{f_{c}^{\prime }}{\varepsilon_{co}}\right)r_{c}\times s_{f}} \end{array}\right.$$

(10) $$R_{sv}=\frac{n_{f}t_{f}E_{f}}{\left(\frac{f_{c}^{\prime }}{\varepsilon_{co}}\right)b}$$

where the confinement stiffness ratio of the FRP in hoop and vertical directions are denoted by *R_sh_* and *R_sv_*, respectively; *w_f_* and *s_f_* (mm) are the width of the FRP strip and center to center spacing between FRP strips in the partial-wrapping system, respectively; *b* is the width of the tension section side.

**Figure 2:**
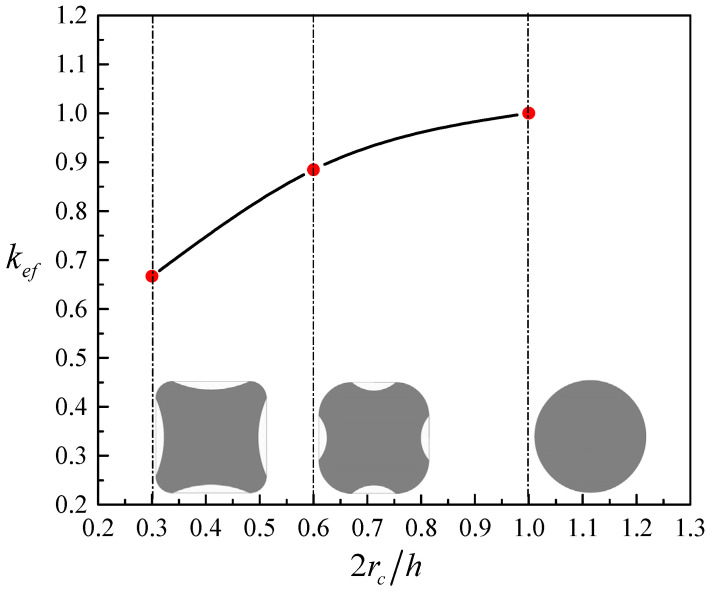
*k_ef_* versus 2*r_c_*/*h*: considering the effect of increasing the corner radius at a constant section width.

**Figure 3:**
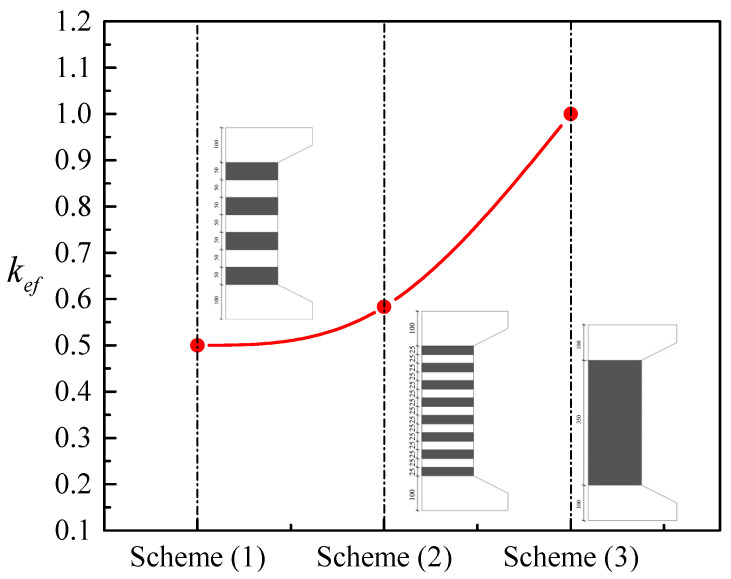
Relationship between different wrapping schemes by Yang et al. [22] and the proposed *k_ef_*.

In the conclusion reached by Pham and Hadi [51], it was assumed that the actual rupture strain of FRP is a function of the ratio of the corner radius to the width of the shorter section side (2*r_c_*/*b*) in addition to the stiffness of the FRP in relation to the unconfined concrete. In their study, the factor *A* = 2*r_c_*/*bR_s_* was finally used. In the current paper, this factor is modified as *A* = 2*r_c_k_ef_*/*hR_sh_*, in which the ratio 2*r_c_*/*b* is replaced by 2*r_c_*/*h*. FRP-confined concrete in rectangular sections have a different response compared with FRP-confined concrete in circular sections, where the stress distribution of FRP wraps is not uniform over the rectangular sections. The nonuniformity of confinement reduces the effectiveness of the FRP confinement. To predict the confined strength of rectangular concrete columns, the shape factor *k_sf_* by Equation (10) is used to consider the reduced strength due to the nonuniformity of the confinement, which was originally suggested by Lam and Teng [2]. The influence of the variation of 2*r_c_*/*h* on the efficiency of FRP confinement is illustrated in Figure 2. In Equation (11), *k_se_*, is to reflect the resulting improvement in the strain of transverse FRP hoops as the width and center-to-center spacing of FRP strips reduced at a constant volumetric ratio of FRP wraps, as investigated by Xian et al. [39] and Yang et al. [22]. Schemes 1 and 2 from the study of Yang et al. [22] are drawn in Figure 3 in relationship with the proposed *k_ef_*. For fully wrapped columns, the second part of *k_se_* is taken to be 1.

(11) $$k_{ef}=k_{sf}\times k_{se}=\left[1-\frac{\left(h/b\right){\left(b-2r_{c}\right)}^{2}+\left(b/h\right){\left(h-2r_{c}\right)}^{2}}{3A_{g}\left(1-\rho_{s}\right)}\right]\left[\frac{\left(N_{s}-1\right)s_{f}}{H_{c}}\right]$$

where *ρ_s_* is the longitudinal steel reinforcement ratio; *N_s_* is the number of FRP strips within the clear height region *H_c_* (mm) (between the end corbels); *A_g_* (mm^2^) is the gross cross-sectional area of a rectangular-section with rounded corners originally proposed by the ACI Committee [59] and can be given by the following expression that has been adopted by a large number of researchers in their models (e.g., Wang et al. [10,11]).

(12) $$A_{g}=bh-\left(4-\pi \right)r_{c}^{2}$$

**Figure 4:**
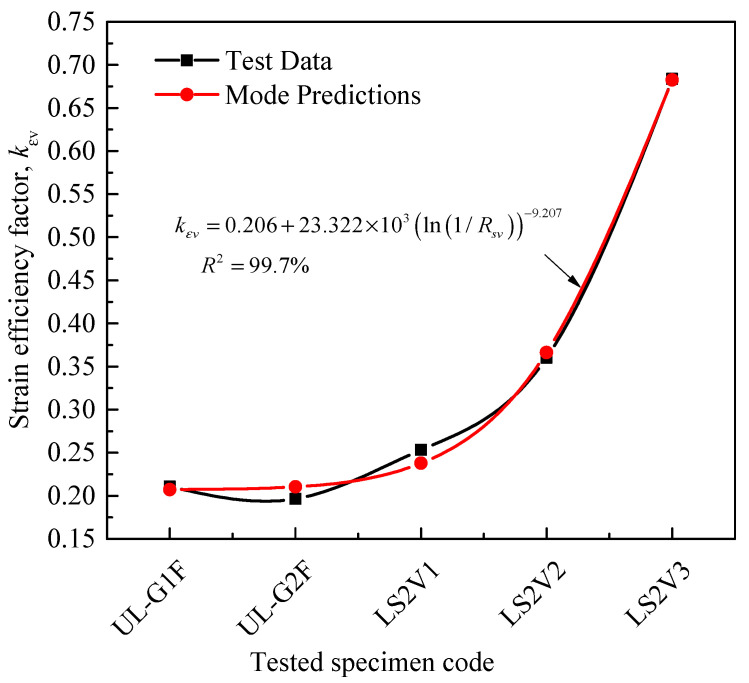
Comparison between experimental and predicted longitudinal FRP strain efficiency factors.

To consider the effects of column parameters on the FRP hoop rupture strain, as discussed in this paper, the first part of Equation (13) is proposed on the basis of a multi-parameter regression analysis of results provided by El Maaddawy et al. [19] and Yang et al. [22] and selected tests of Lin et al. [43]. Because of the limited test results used in the proposed expression, limits based on results of Wang et al. [10,11] for the calculated *k_εh_* were also employed. Based on results from the studies of Hassan et al. [21] and Yang et al. [22], the rupture strain of longitudinal FRP sheet was also determined using the proposed Equation (14), in which the FRP rupture strain of longitudinal CFRP sheet in tensile zone increases linearly with an incremental increase of CFRP layers, as indicated by Yang et al. [22]. As a result, Equation (14) seems to be composed of two ranges of linear relationships for the database with two types of FRP-wrapped specimens: carbon FRP-wrapped specimens (CFRP, 3 specimens) and glass FRP-wrapped specimens (GFRP, UL-G1-F & UL-G2-F). This can be clearly seen in Figure 4, in which the two GFRP-wrapped specimens have the lowest strains compared with the higher trend of CFRP results. Based on CFRP results of Yang et al. [22], *B*_4_ in Equation (14) is the maximum ratio of longitudinal strain to the ultimate strain from coupon tests and is equal to 0.678. The values of the parameters in this subsection (i.e., B1 to B6 in Equation (13)) and other discussions, which are obtained using a multi-parameter regression analysis, are summarized in Table 4.

(13) $$k_{\varepsilon h}\left\{ \begin{array}{l} \max\left(\left(B_{1}+B_{2}\times \ln\left(\frac{2r_{c}/h}{{\left(R_{sh}\right)}^{B_{3}}}\times k_{ef}\right){\left(B_{4}+\frac{e}{h}\right)}^{B_{5}}\right),B_{6}\right)\;\\ if\left(\left(\frac{e}{h}\right)\leq 0.326,k_{\varepsilon h}=B_{6},h\geq 300\;\mathrm{mm}\right);k_{\varepsilon h}=0.4\\ if\left(\left(\frac{e}{h}\right)\leq 0.326,k_{\varepsilon h}=B_{6},h<300\;\mathrm{mm}\right);k_{\varepsilon h}=0.6 \end{array}\right.$$

(14) $$k_{\varepsilon v}=\max\left[B_{1}+B_{2}{\left(\log\left(1/R_{sv}\right)\right)}^{B_{3}},B_{4}\right]$$

**Figure 5:**
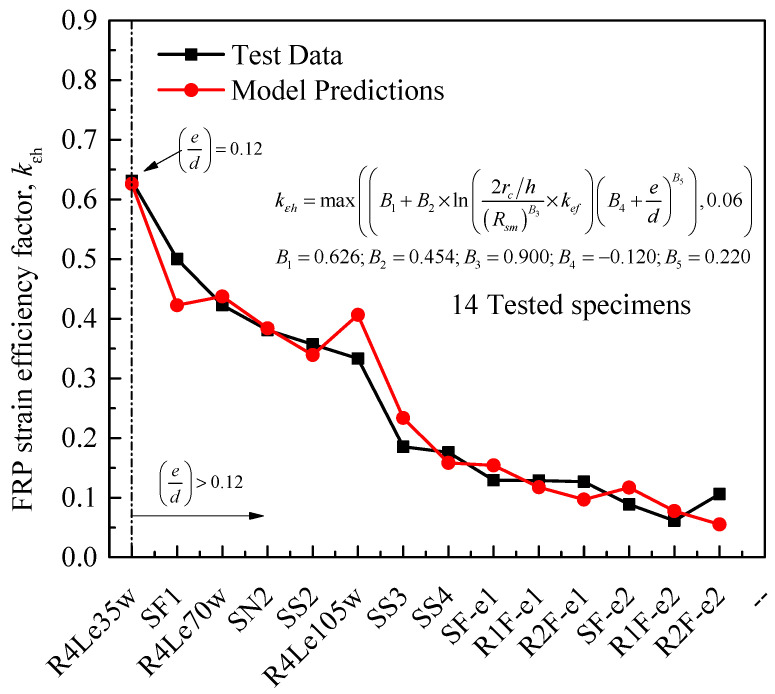
Comparison between experimental and predicted hoop FRP strain efficiency factors.

The experimental and theoretical results of the rupture strains of CFRP wraps are compared in Table 5. A total of 9 models from Barrington et al. [54], Wang et al. [11], Song et al. [28], Ozbakkaloglu [60], Hadi et al. [51], Hany et al. [61], Wang et al. [62], ACI 440.2R [63,64], Yang et al. [22] were investigated in Table 5. The comparison between the predictions and test results shows the accuracy of current and existing models in calculating the strain efficiency factors. Over the range of parameters considered in the tests (i.e., eccentricity ratio, *e*/*h*) (Table 2), the comparisons reveal that the tested hoop rupture strain results are significantly overestimated by the summarized models. Comparisons to evaluate the performance of the proposed expressions are also provided in Figure 4 and Figure 5. Among the provided models, the model of this paper has the highest correlation between the predictions and test data (the averaged ratios of model predictions to the test data are 99.95 and 100%, respectively, for *k_εh_* and *k_εv_*). In addition, the errors of these models were verified. Although the model of Barrington et al. [54] gives an average ratio of the model predictions closer to the test data, the estimated errors of this model are higher compared to those of the current model.

Recognizing that pure compressive loading is impossible in practice, RC columns are commonly designed to account for a minimum load eccentricity (Wang et al. [65]). Note that ACI [66] requires a minimum eccentricity of 10% for tied columns and recommends a further reduction. The conclusion drawn by Mirmiran et al. [67] is that the maximum eccentricity is within 10–12% of the section width. It was also confirmed by others, i.e., Bisby and Ranger [68], that minimum eccentricities are necessary for members, and that FRP-wrapped RC columns member reduction factors should be reduced further. Indeed, this is one possible justification for the 0.95 reduction factor that is currently included in the ACI 440.2R-08 [64] design procedures. The results of the hoop rupture strain from the proposed model, as provided in Figure 5, confirms a minimum eccentricity of about 0.12 *h*, which is similar to that of Mirmiran et al. [67] (identified in this analysis as *B*_4_ = −0.12) for FRP-confined RC columns. The expressions are calibrated based on limited tests because no other results are reported for FRP-confined rectangular RC columns. Further research taking into account a wider range of test parameters may be necessary to check the accuracy of the model.

### 3.2. Modeling of Maximum Axial Capacity

#### 3.2.1. Equations for Unconfined RC Columns

**Figure 6:**
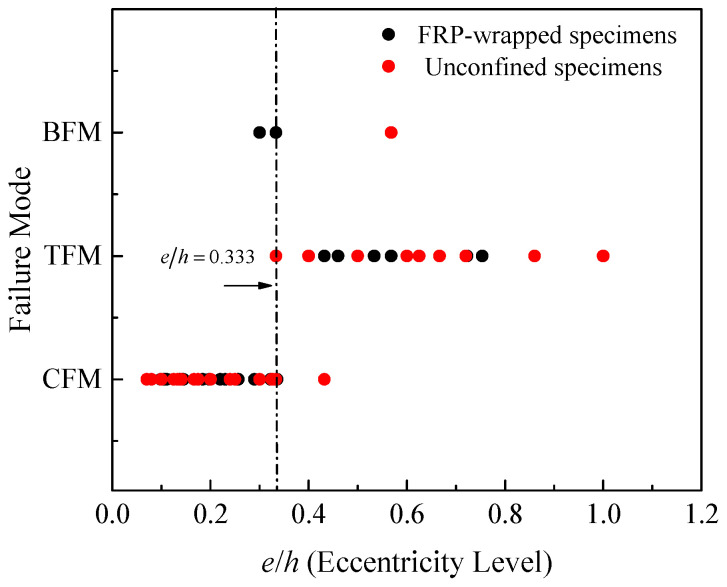
Eccentric load ratio via failure mode.

**Figure 7:**
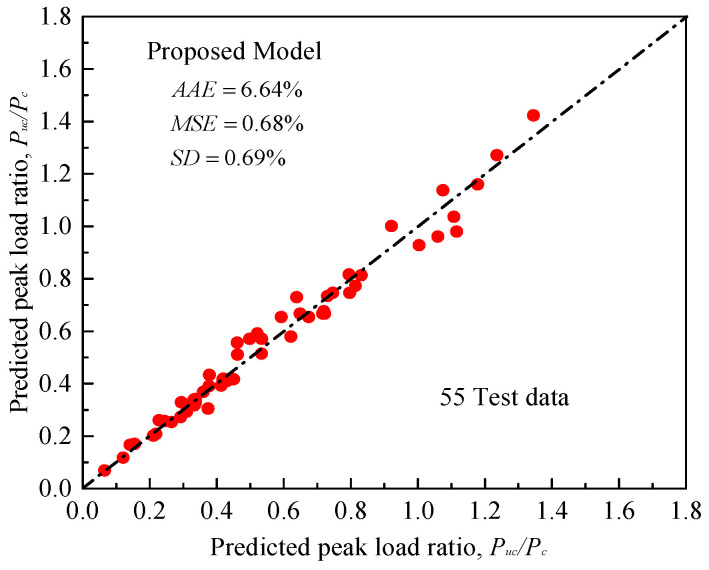
Comparison between experimental and predicted peak load ratios for columns with internal steel confinement.

**Figure 8:**
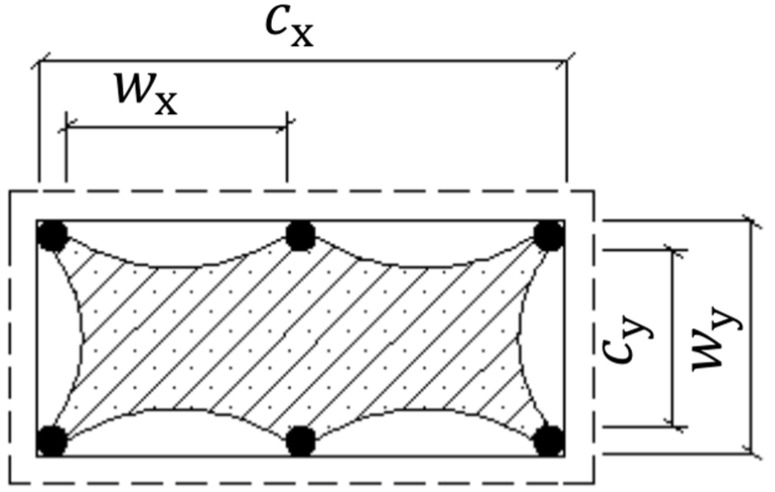
Parameters of effectively-confined concrete area by steel hoops.

Generally, the tests summarized in Table 2 indicated that most of the specimens tested at an eccentricity ratio of about 0.33 experienced compression failure (see Figure 6), while the failure of specimens at larger eccentric loads was defined as transition or flexural. In this subsection, predictive expressions are proposed to better estimate the maximum strength of FRP-confined rectangular RC columns. The expressions of the model are derived by utilizing the experimental data of specimens summarized in Table 2. A total of 142 test results were taken into account during the regression analysis. A total of 55 out of these results were for columns confined with internal steel hoops, and the remaining results were for FRP-confined rectangular RC columns. Equation (15) can be used to determine the increase in the maximum load capacity of RC columns relative to the strength of unconfined concrete cylinders based on a multi-parameter regression analysis and interpretation of the experiments. The correlation coefficients (*R*^2^) are about 93.8% and 94.4% for the first and second parts of the expression, respectively. The parametric form of the expression is provided in Equation (15), which is similar to that of Song et al. [28] but with several modifications. One of these is that the maximum load was considered to be related to the load of unconfined cylinders (*P_c_* = $f_{c}^{\prime }$(*A_g_* − *A_s_*)) to estimate the contribution of steel confinement. To evaluate the accuracy of the proposed expression, the results and the estimated errors are provided in Figure 7. The expression takes into account the effects of strength of unconfined concrete and hoop and longitudinal steel reinforcement. Relative to the unconfined concrete strength, the dimensionless factors to consider the contribution of steel reinforcement are given in Equations (16) and (17), respectively.

(15) $$\frac{p_{uc}}{P_{c}}\left\{ \begin{array}{l} if(0.10\leq e/h\leq 0.33)\left[1+B_{1}k_{es}{\left(\lambda_{sh}\right)}^{B_{2}}+B_{3}{\left(\lambda_{sv}\right)}^{B_{4}}\right]\\ \;\times {\left(1+\frac{e}{d}\right)}^{B_{5}}\left(1-B_{6}\times e^{{\left(f_{c}^{\prime }\right)}^{B_{7}}}\right)\\ if(0.40\leq e/h\leq 1.00)B_{1}+\left[1+{\left(\frac{e}{h}\right)}^{B_{2}}\left(\lambda_{sv}-B_{3}{\left(\lambda_{sv}\right)}^{B_{4}}\right)\right]\\ \;\times {\left(1+\frac{e}{h}\right)}^{B_{5}}\left(1-B_{6}\times e^{{\left(f_{c}^{\prime }\right)}^{B_{7}}}\right) \end{array}\right.$$

(16) $$\lambda_{sh}=\frac{\rho_{eh}f_{yh}}{f_{c}^{\prime }}$$

(17) $$\lambda_{sv}=\frac{\rho_{s}f_{yl}}{f_{c}^{\prime }}$$

where *f_yh_* and *f_yl_* (MPa) are the yield strengths of hoop and longitudinal steel reinforcement, respectively; the coefficient *k_es_* is used to quantify the effective confinement induced by the steel hoops in the lateral and vertical directions. This coefficient can be calculated by using the following expression originally developed by Sheikh and Uzumeri [69], in which *c_x_* and *c_y_* (mm) are the concrete core dimensions to the centerline of the peripheral hoop reinforcement (Figure 8), and herein replaced by $dxy=\sqrt{b^{2}-h^{2}}\left(\mathrm{mm}\right)$; *s*′ (mm) = clear spacing between the steel hoops; *ρ_cc_* is the ratio of the longitudinal reinforcement area divided by the area of the inner concrete core (*ρ_cc_* = *A_s_*/*c_x_* × *c_y_*).

(18) $$k_{es}=\frac{1-\sum \left(w_{xi}^{2}+w_{yi}^{2}\right)/6c_{x}c_{y}}{1-\rho_{cc}}\;\times {\left(1-\frac{s^{\prime }}{d_{xy}}\right)}^{2}$$

The effective volumetric ratio of hoop steel reinforcement, *ρ_eh_*, is derived from Equation (17).

(19) $$\rho_{eh}=\frac{\rho_{sx}c_{x}+\rho_{sy}c_{y}}{c_{x}+c_{y}}$$

(20) $$\rho_{sx}=\frac{A_{shx}}{sc_{x}}$$

(21) $$\rho_{sy}=\frac{A_{shy}}{sc_{y}}$$

where the terms *A_shx_* and *A_shy_* (mm^2^) are the total cross-sectional areas of the hoop steel bars in the longer and shorter sides, respectively; *c_x_* and *c_y_* (mm) are the distances between the center-lines of the perimeter hoop in the longer and shorter sides, respectively (see Figure 8).

**Figure 9:**
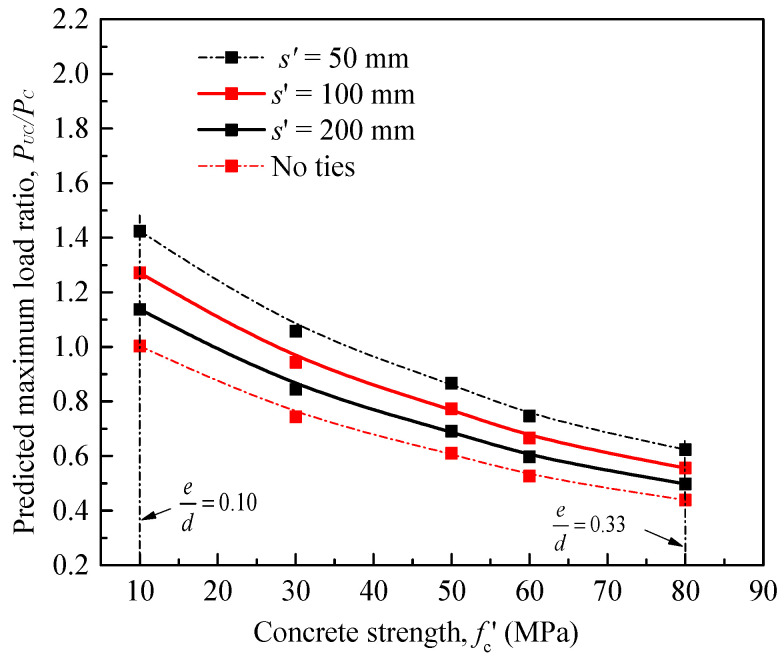
Relationship between the relative load ratio and the concrete compression strength for specimens with different steel hoops and small eccentricity ratios.

The first part of Equation (15) (i.e., 0.10 ≤ *e/h* ≤ 0.33) was utilized for a parametric study to identify the effects of strength of unconfined concrete and steel confinement on the maximum compression load of RC columns. The specimens were with material and geometry details similar to those used by Jaturapitakkul et al. [27] but with varying unconfined concrete strength and steel confinement: One specimen was without internal hoop reinforcement (i.e., no ties) and three specimens with different hoop steel spacing (i.e., *s* = 50, 100, and 200 mm) were considered for the interaction between the load eccentricity and the unconfined concrete strength. The predicted ratios of the relative strength (*P_uc_*/*P_c_*) and the strength of unconfined concrete ($f_{c}^{\prime }$) ranging between 10 and 80 MPa are shown in Figure 9. It is generally evident that the relative strength increases as the spacing of the steel hoops reduces. Although the relative strength ratio is seen to be higher at small values of $f_{c}^{\prime }$, it remains essentially constant at large $f_{c}^{\prime }$ values, irrespective of the different amount of steel confinement. This confirms that the increase of the confined concrete strength is more significant for RC members with normal-strength concrete rather than high-strength concrete (e.g., Vincent and Ozbakkaloglu [70], Eid et al. [71], Wu et al. [72]). The effectiveness of steel-confinement in eccentrically loaded RC columns is also influenced by the increase of the load eccentricity ratio. When the *e/h* value is equal to 0.3, the load capacity is found to be mostly dependent on the concrete compressive strength and tensile steel bars (e.g., Daugevičius et al. [73]). In this paper, for the range 0.40 ≤ *e*/*h* ≤ 1.00, the *P_uc_*/*P_c_* results were predicted using the second part of Equation (15), without considering the steel confinement.

**Figure 10:**
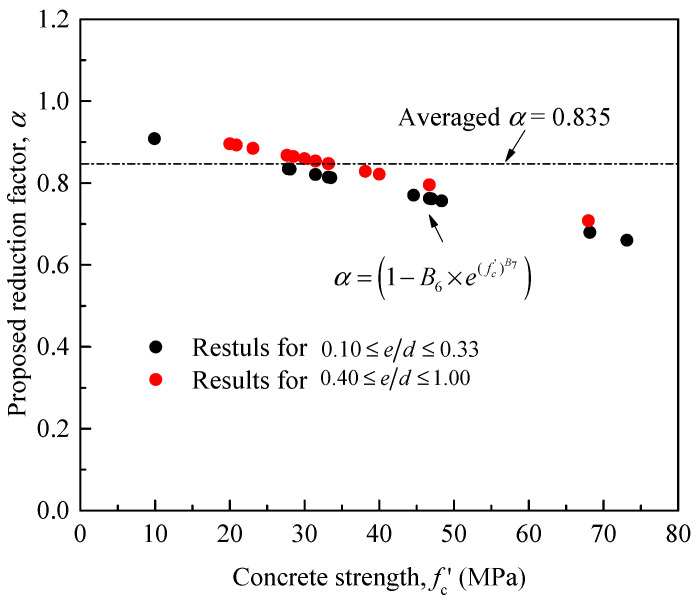
Relationship between the calculated reduction factor, *α*, and the unconfined concrete compression strength for the unconfined RC specimens summarized in Table 2.

The ACI Committee (318-14) [74] reported an expression (*N_uo_* = $f_{c}^{\prime }$(*A_g_* − *A_s_*) + *f_yl_A_s_*) for the pure axial compressive load of an unconfined RC column. The factor *α* is defined as the ratio between strength of concrete casted in place to the strength of concrete cylinder. The *α* value for compressive concrete with strength up to 50 MPa is equal to 0.85, which is also similar to that used by the ACI [75]. The Australian Code [76] suggested a value of *α* within the limits 0.72 ≤ *α* ≤ 0.85, whereas the Canadian standards [77] recommended the limit 0.67 ≤ *α* ≤ 0.80. As for the eccentrically tested RC columns (Table 2), Figure 10 shows almost closed values of *α* predicted using Equation (15). In general, the predicted results indicated the increase in the unconfined concrete strength does not show any change in the trends when the eccentricity ratio increases; using the first part of Equation (15), the minimum value of *α* calculated for a concrete strength of 73.13 MPa (i.e., specimens tested by Hadi et al. [18]) is 0.66, while the maximum value of *α* for concrete with strength of 9.92 MPa (i.e., specimens tested by Jaturapitakkul et al. [27]) is 0.91; using the second part of Equation (15), the minimum value of *α* for a concrete strength of 67.98 MPa (i.e., specimens tested by Sadeghian et al. [36]) is 0.71, while the maximum value of *α* corresponding to an $f_{c}^{\prime }$ value of 20 MPa (i.e., specimens tested by El Maaddawy et al. [19]) is 0.90. The averaged *α* value for all RC specimens is 0.84, which is almost similar to that of the ACI [64]. This can confirm a satisfactory accuracy of the analysis of this paper.

**Figure 11:**
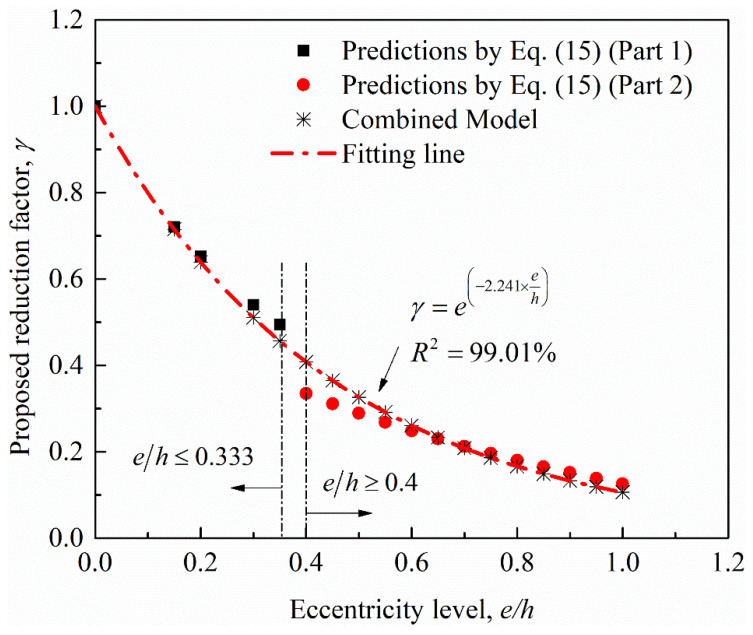
Relationship between the calculated reduction factor and the eccentricity ratio for the unconfined RC specimens summarized in Table 2.

Further confirmation is the strong correlation when the trends of the reduction due to the increase of eccentricity are combined into one equation, as shown in Figure 11, in which the *R*^2^ value is obtained as 99%. Table 6 also presents a clear comparison between reductions in strength obtained from tests and the proposed expression for a total of 15 RC specimens tested by five independent research groups (Jinglong et al. [42], Darby et al. [33], Saljoughian and Mostofinejad [37], El Maaddawy et al. [19], Yang et al. [22]). The comparisons show that the model is able, with good accuracy, to estimate the reduced increment of unconfined peak strength. The average absolute error, *AAE*, is about 6.6% and the averaged ratio of model predictions to test data is 95.3%.

#### 3.2.2. Equations for FRP-Confined RC Columns

Tests on the effect of eccentricity on the effectiveness of FRP confinement in enhancing the axial strength of reinforced columns have confirmed that using FRP wraps can provide significant strength enhancement (e.g., [29,36,68,73,78,79,80,81]). The enhancement was found to be significant for members experienced compression control failure (e.g., [82,83]). Columns vertically-reinforced and laterally wrapped with FRP exhibited further strength enhancements even in tests of large eccentric load ratios (e.g., Al-Nimry and Soman [83], Al-Nimry and Al-Rabadi [84], Al-Nimry and Neqresh [32]). In these studies, a negative effect of the increase in load eccentricity on peak strength of FRP-confined columns was noticed.

**Figure 12:**
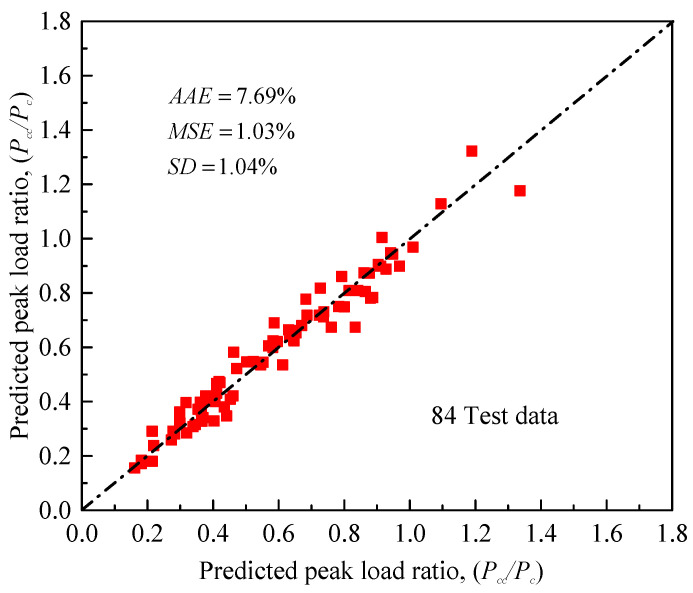
Comparison between experimental and predicted peak load ratios for FRP-confined RC columns.

However, only few of the existing models have considered the effect of load eccentricity on the behavior of FRP-confined concrete (e.g., Hu et al. [30], Wu and Jiang [85], Fahmy and Farghal [86], Wu and Cao [87], Cao et al. [88]). No model that considers the positive effect of the two wrapping configurations (i.e., horizontal and vertical FRP sheets) and the internal steel confinement on axial load capacity has been reported earlier for FRP-confined rectangular reinforced columns. In this subsection, Equation (20) for predicting the peak strengths is therefore introduced based on the analysis and interpretation of the experiments in Table 2. The model considers the contribution of FRP wraps and internal steel reinforcement as well as the negative effects of increased eccentricity and strength of unwrapped concrete, as considered by Song et al. [28]. The correlation coefficient (*R*^2^) is finally obtained as 94.5%. As shown in Figure 12, comparisons of the analytical and experimental test values generally reveal a very good accuracy, with insignificantly estimated errors.

(22) $$\frac{p_{cc}}{P_{c}}=\left[0.75\times \left(\frac{p_{uc}}{P_{c}}\right)+\left(B_{1}\lambda_{fv}{}^{B_{2}}+B_{3}k_{ef}{}^{B_{4}}\lambda_{fh}{}^{B_{5}}\right){\left(1+\frac{e}{h}\right)}^{B_{6}}\right]\left(1-B_{7}f_{c}^{\prime }\right)$$

where the contribution of internal steel reinforcement determined by the previously proposed Equation (15) for unconfined RC columns was partly considered in Equation (22). The terms *λ_fv_* and *λ_fh_* are introduced as dimensionless parameters to consider the contributions of the vertical and horizontal FRP wraps to the enhancement of the peak stress. Respectively, these parameters are proposed as

(23) $$\lambda_{fh}=\left\{ \begin{array}{l} \frac{n_{f}t_{f}E_{f}\varepsilon_{fu}}{f_{c}^{\prime }D}\\ \frac{n_{f}t_{f}w_{f}E_{f}\varepsilon_{fu}}{f_{c}^{\prime }D\times s} \end{array}\right.\left(\mathrm{For}\;\mathrm{full}\;\mathrm{and}\;\mathrm{partial}\;\mathrm{wrapping},\;\mathrm{respectively}\right)$$

(24) $$\lambda_{fv}=\frac{n_{f}t_{f}E_{f}\varepsilon_{fu}}{f_{c}^{\prime }b}$$

**Figure 13:**
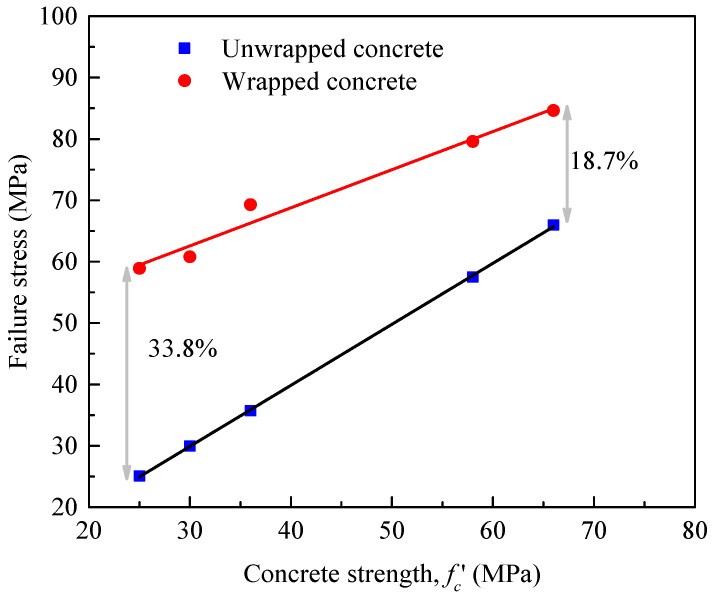
Normalized peak compressive axial load versus load eccentricity.

Previous research on pure compressive tests has studied the importance of shear failure wedges formed in FRP-wrapped cylinders at higher load levels (i.e., Mohamed et al. [89]); the development and also movement of the shear failure wedges is shown to be different in concretes of varied strength of unconfined concrete. From the results of Bisby et al. [90], as provided in Figure 13, for FRP-confined concrete cylinders, large enhancement in their strengths was achieved for different levels of concrete strengths by FRP wraps; the FRP wraps reduced the dilatancy of all strengths of concrete at all load levels. Confinement by FRP wraps resulted in a greater enhancement in strength for low strength concrete, with an increase of about 33.8%. A similar finding was also reported by Mandal et al. [91]. As the unconfined compressive strength increased from 25 to 66 MPa, the increment of enhancement in FRP-confined strength was reduced to 18.7% (a reduction of about 44.8%).

**Figure 14:**
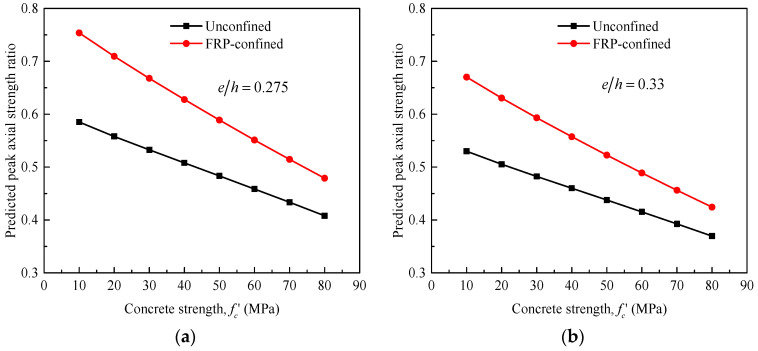
Predicted peak load ratios for unconfined and FRP-confined RC columns versus concrete strength: considering the effect of increased eccentricity ratio and concrete strength. (**a**) For *e*/*h* = 0.275 and (**b**) For For *e*/*h* = 0.33.

**Figure 15:**
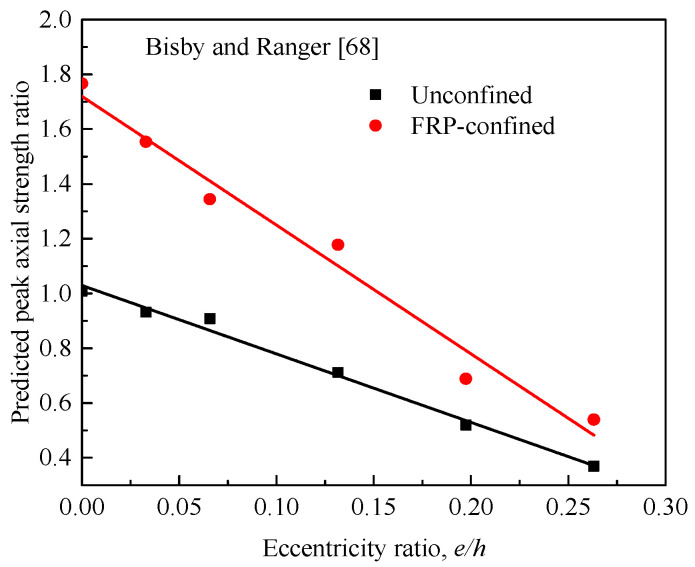
Normalized peak compressive axial load versus load eccentricity (Bisby and Ranger [68]).

As for the eccentric tests of this paper, a specimen denoted as SS3 confined with three layers of FRP wraps was selected from the tests of Yang et al. [22] for a parametric study to identify the effects of unconfined concrete strength ($f_{c}^{\prime }$) and eccentricity ratio (*e*/*h*) on the peak compression load of FRP-confined RC columns. For a clear investigation, the model predictions were provided for unconfined and FRP-confined concrete, with the same amount of steel and FRP confinement. Similar to the trends reported by Bisby et al. [90], Figure 14 also confirms linear relationships obtained between the predicted columns strength ratios (*P_uc_*/*P_c_* and *P_cc_*/*P_c_*) and the compressive strength of unconfined concrete in the range between 10 MPa to 80 MPa. The overall effects of unconfined concrete strength, eccentricity ratio, and FRP wrapping are clear. The strength ratio decreases as $f_{c}^{\prime }$ and *e*/*h* increases. Figure 14 also shows that the reduction in strength of FRP-wrapped RC columns is proportionally higher than that of unwrapped column, as evidenced by the steeper slope of the trend. In terms of the effect of increased eccentricity ratio, typical observations for FRP-confined circular RC columns have been recently drawn by Bisby and Ranger [68], as shown in Figure 15; noting that Al-Nimry and Al-Rabadi [84] suggested that reductions in strengths with the increase of eccentricity ratio were less pronounced for the FRP-wrapped specimens than for the unwrapped ones.

As already stated in the Introduction, only limited studies on the effect of internal steel ties are available in the technical literature. Of these pure concentric tests, Wang et al. [10,11] investigated the impact of variation in steel tie spacing on the column behavior of a medium with dimensions 204 mm × 204 mm × 612 mm and large-sized 305 mm × 305 mm × 915 mm square RC columns confined with different numbers of CFRP layers (i.e., *n_f_* = 1, 2, 3). The hoop steel reinforcement ratios were 0% for plain concrete and 0.5% and 1% for concrete reinforced with an inadequate or normal amount of steel ties, respectively. The center-to-center hoop spacings that correspond to 0.5% and 1% ratios were 80 mm and 40 mm for specimens of the large size, and 120 mm and 60 mm for the medium-sized specimen. The averaged strength of unconfined compressive cylinders obtained at 28 days and the time of the actual test was about 25 MPa. The study generally concluded that the internal steel ties contributed to the enhancement of the peak axial strength of FRP-confined RC columns. In this paper, a deep inspection of their test results revealed that the strength of confined RC specimens is either higher than the unconfined RC specimens or in the same range, except for specimens with a low level CFRP confinement. Almost similar results were also reported in a recent study by Doan et al. [92] and Al-Nimry and Neqresh [32]. For instance, from the test program of Wang et al. [10,11], the square unwrapped (denoted as L0) reinforced (denoted as H1 and H2) S1H1L0 and S1H2L0 specimens of larger size and specimens S2H1L0 and S2H2L0 of medium size have the same unconfined concrete strength and longitudinal reinforcement ratio. As the tie spacing reduces from 80 mm to 40 mm for specimens S1H1L0 and S1H2L0 and from 120 mm to 60 mm for specimens S2H1L0 and S2H2L0, the strength at peak increased by almost 8.1% and 8.7%, respectively. As for the medium-sized specimens wrapped with one, i.e., S2H1L1M and S2H2L1M or two CFRP layers, i.e., S2H1L2M and S2H2L2M, the confined strength was, in contrast, decreased by about 7% and 0.87%, respectively, as the spacing between the steel ties reduced. A larger reduction of about 12.5% in peak strength was for the large-sized specimens confined with one CFRP layer, i.e., S1H1L1 and S1H2L1. Almost similar strengths, with an increase of 1.72% and 0.81%, were achieved when the specimens were wrapped with two and three layers of CFRP wraps and the tie spacing reduced from 80 mm to 40 mm, respectively. Irrespective of the specimen size, the impact of variation in the amount of internal steel ties on FRP-confined strength are generally lower than those of the unwrapped ones.

**Figure 16:**
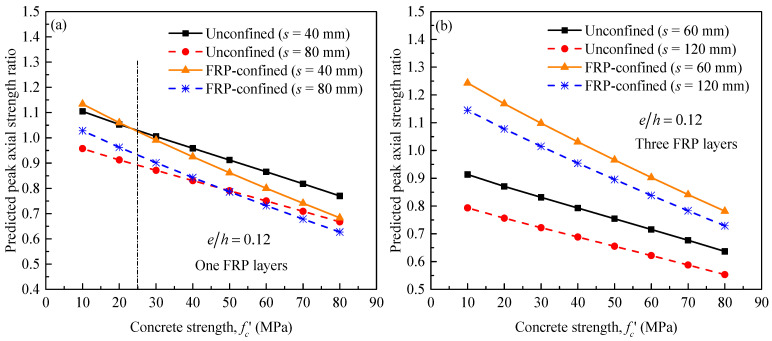
Predicted peak load ratios for unconfined and FRP-confined RC columns versus concrete strength: considering the effect of the effects of variation in internal steel confinement. (**a**) For one FRP Layer (**b**) For three FRP Layers.

To confirm the accuracy of the proposed model, the same example as provided in Figure 14 was also used in Figure 16 for a parametric study, to study the positive impact of increasing the ratio of internal steel ties for columns with unconfined concrete strength that varied between 10 and 80 MPa on the peak strength of unconfined concrete and FRP-confined reinforced concrete. The tie spacings, i.e., *s* = 40 mm and 80 mm and the numbers of CFRP layers, i.e., *n_f_* = 1, provided in Figure 16a are similar to those of Wang et al. [10,11]. In addition, for reasonable comparisons, a minimum eccentric loading ratio of 0.12 is applied to represent their concentric tests. Regardless of the level of internal confinement, the enhancements in strength of FRP-confined columns are generally lower than those of the unwrapped columns. For a case where the strength of unconfined concrete is similar to that of Wang et al. [10,11], the trend-line of the unwrapped specimen with tie spacing of 40 mm meets with the trend-line of the confined specimen with the same tie spacing, which indicates that the enhancement in strength was typically achieved by either confinement by steel ties or confinement provided by using both steel ties and FRP wraps. For the unwrapped ones, the two trend-lines of the 80-mm tie-spaced specimens were identical at larger values of unconfined concrete strength, i.e., $f_{c}^{\prime }$ = 50 MPa, which indicates a lower confinement was achieved compared with that of the 40-mm tie-spaced specimens. Irrespective of the lower effect of increased volumetric ratio of steel ties on the response of FRP-confined concrete compared to that of the unconfined concrete, a higher strength enhancement was achieved for the three-layered column when the tie spacing reduced from 120 mm to 60 mm (Figure 16b). Similar results were also reported by others (e.g., Al-Nimry and Neqresh [32]).

The decreased level of enhancement in peak strength of FRP-confined RC columns as a function of the increase of eccentricity ratio is listed in Table 7. Results obtained from tests and the proposed model for 40 FRP-confined RC specimens tested by Jinglong et al. [42], El Maaddawy [19], Sadeghian et al. [36], El Maaddawy et al. [29], Darby et al. [33], Hadi et al. [18], Hassan et al. [21], Elsayed et al. [40] and Shaikh and Alishahi [34] are presented. These comparisons cover a wide range of eccentric load ratios ranging between 0.2 and 1. It can be generally seen that the proposed model gives very good predictions for the reduced increment of the FRP-confined peak strength. The *AAE* value is calculated as 9.96% and the averaged ratio of model results to the experimental results is 100.4% within the limit specified by literature [93].

### 3.3. Modeling of Axial Capacity at Failure

#### 3.3.1. General

Concrete columns sufficiently confined using FRP wraps can exhibit an ascending load-strain response. This is widely-defined as the sufficiently confined concrete. In such a case, significant enhancements in their axial strength can be achieved. Otherwise, for a case where the amount of internal or external confinement is not sufficient, the columns exhibit a descending response. In other terms, if the calculated *P_uc_*/*P_cc_* ratio is greater than 1, such a threshold represents the sufficiently confined concrete, and conversely, when the value of *P_uc_*/*P_cc_* is less than 1, the columns exhibit a descending response. In reality, the columns need to be accurately designed to obtain sufficient confinement (e.g., [10,14,51]). The strength of the confined columns at failure is an important parameter for designing a sufficient confinement level. In the following discussion, on the basis of a statistical interpretation of the tests in Table 2 and observations of other existing studies, new expressions are thus proposed to accurately predict the failure strength of FRP-confined RC columns.

#### 3.3.2. Equations for Unconfined RC Columns

Tests on concentrically loaded unwrapped RC columns revealed that the strength at failure condition is found to be 80% of the peak strength (e.g., Wang et al. [10]). Primarily based on the results of 19 eccentrically loaded unwrapped RC columns from the tests reported in Table 2, a strong correlation was also found between the ultimate and the peak strength ratios, as reflected in the following proposed relationship with an (*R*^2^) value of about 98%. The tested and predicted results are provided in Table 8, in which the averaged ratio of the ultimate strength to the peak strength is almost close to the result of Wang et al. [10]. The average absolute error is about 5.15% and the average ratio of the model predictions to the test results is 99.4%.

(25) $$\left(\frac{p_{uc}^{u}}{P_{c}}\right)=B_{1}\left(\frac{P_{uc}}{P_{c}}\right){\left(\frac{e}{d}\right)}^{B_{2}}+B_{3}{\left(\frac{P_{uc}}{P_{c}}\right)}^{B_{4}}\left(\frac{e}{d}\right)$$

where the peak strength ratio *P_uc_*/*P_c_* can be calculated using Equation (15); the parameters obtained from the analysis are provided in Table 4.

#### 3.3.3. Equations for FRP-Confined RC Columns

During analysis, it was found that the level of enhancement in axial strength at failure due to FRP confinement does not appear to be influenced by the variation in unconfined compression concrete strength, as also confirmed by Bisby et al. [90]. With regard to the FRP reinforced column response, using longitudinal and lateral FRP reinforcement allowed columns to resist higher axial loads while increasing their flexural rigidity compared to columns wrapped only with FRP hoop (e.g., Al-Nimry and Al-Rabadi [84]). The enhancement in ultimate strength due to the use of FRP hoop or both the FRP hoop and longitudinal reinforcement is carefully accounted for, and eight specimens from the test programs of Hassan et al. [21] and Al-Nimry and Neqresh [32] that exhibited different levels of strength enhancements at peak and failure loads are singled out for deeper inspection. Results of these specimens are selected and studied for additional reasons, namely (1) they had varying dimensions of cross sections; (2) they had different ratios of internal longitudinal and hoop steel reinforcement; (3) they were with different FRP materials and numbers of FRP layers and tested under different eccentric ratios. In small eccentric tests by Hassan et al. [21], GFRP wraps were applied in single or double layers; in partial or full wrapping, whereas, in large eccentric tests, longitudinal GFRP layers were applied and overlaid by a GFRP hoop partially applied in a single layer. In the tests of Al-Nimry and Neqresh [32], the wraps were fully applied in a single layer; and an additional important point is that columns with high-strength concrete were studied by Hassan et al. [21], whereas normal-strength concrete columns were used in the study of Al-Nimry and Neqresh [32].

Compared to US-G1F laterally wrapped with one GFRP layer and tested under eccentricity ratio of 0.125, an enhancement of the peak load with 22.3% is obtained for specimen US-G2F by applying an additional GFRP layer. For their counterparts that were reinforced with an additional vertical GFRP layer and tested under larger eccentricity of 0.625, a lesser enhancement of 8.1% is provided. However, there is a larger increase of the ultimate load of 25.2% compared with enhanced peak strength results. Similar results can be also seen for the specimens C50-LC and C65-LC from the tests of Al-Nimry and Neqresh [32], in which the reduction in the ultimate strength due to the increase of eccentricity ratio from 0.25 to 0.325 was lower than those of the C50-C and C65-C wrapped only with FRP hoop. In general, these comparisons are consistent with the conclusions drawn by Hassan et al. [21] as (1) wrapping small eccentrically loaded specimens with two full GFRP layers is the most appropriate way to obtain significant enhancement in strength, and (2) using longitudinal GFRP laminates overlaid by GFRP hoop is a successful choice in large eccentricity loaded columns.

**Figure 17:**
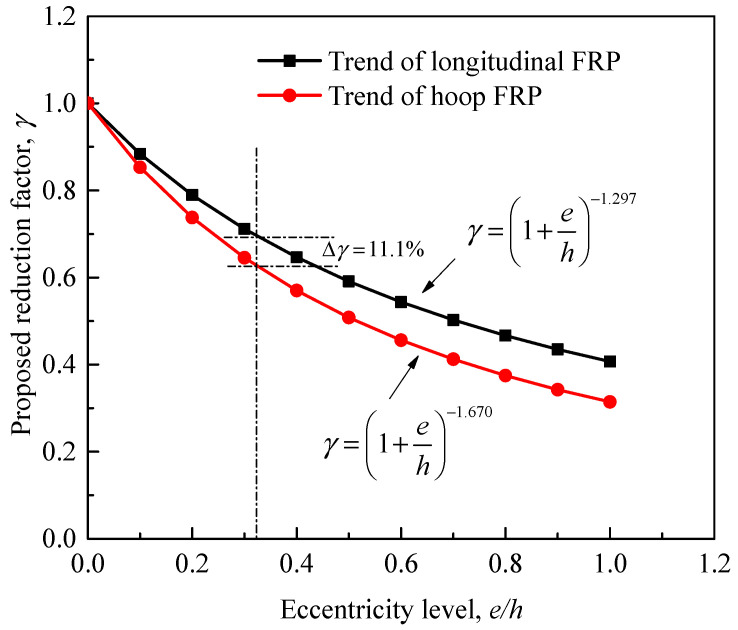
Proposed trends considering a negative effect of increasing eccentricity for columns with FRP hoop only or lateral and longitudinal FRP reinforcement.

**Figure 18:**
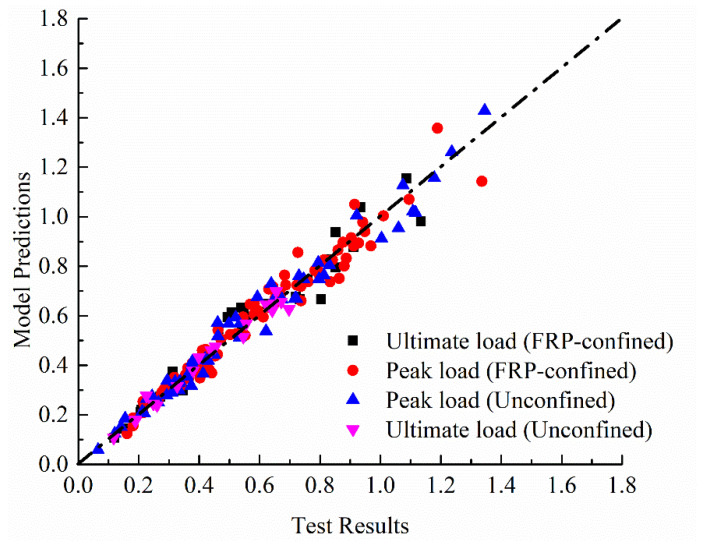
Comparison between experimental and predicted ultimate load ratios for FRP-confined RC columns.

In addition to the contributions made by the internal longitudinal and hoop reinforcement to the strength enhancement, those resulted by using different wrapping systems were carefully considered based on the above-discussed results. Two different equations that can reflect the varied effects of the increased eccentricity ratio on the ultimate strength trends of specimens with longitudinal FRP sheets confined by hoop sheets and specimens confined with FRP hoop sheets only were proposed, as shown in Figure 17. As can be clearly seen, the gap between the two trend lines increases as the eccentricity ratio increases. This proposal confirms the significant role of the use of longitudinal FRP sheets in providing higher improvement in the column response. An example from the results in Table 9 shows that the confined ultimate strengths of 65LC reinforced with longitudinal and hoop FRP sheets was enhanced by 12.9% compared to the 65-C that was confined only with FRP. With an acceptable accuracy, the predictions of the proposed trends in Figure 17 show that the difference in their strength is almost 11.1%. The total enhancement in strength was finally calculated by the following equation. It can be seen from Figure 18 that, in general, the model predictions are in good agreement with the current results as well as the other data. The averaged ratio of tested to predicted strengths for a total of 53 FRP-confined specimens is 102.2%, while the average absolute error and standard deviation are 9.68% and 1.73%, respectively.

(26) $$\frac{p_{cc}^{u}}{p_{c}}=\frac{p_{uc}^{u}}{p_{c}}+B_{1}{\left(\lambda_{fv}^{*}\right)}^{B_{2}}{\left(1+\frac{e}{h}\right)}^{B_{3}}+B_{4}{\left(\lambda_{fh}^{*}\right)}^{B_{5}}k_{ef}{\left(1+\frac{e}{h}\right)}^{B_{6}}$$

where the terms $\lambda_{fv}^{*}$ and $\lambda_{fh}^{*}$ are introduced to consider the contributions of the longitudinal and horizontal FRP layers to the enhancement of the strength at failure and determined by Equations (27) and (28), respectively.

(27) $$\lambda_{fh}^{*}=\left\{ \begin{array}{l} \frac{n_{f}t_{f}E_{f}\varepsilon_{fe}}{f_{c}^{\prime }D}\\ \frac{n_{f}t_{f}w_{f}E_{f}\varepsilon_{fe}}{f_{c}^{\prime }D\times s} \end{array}\right.\left(\mathrm{For}\;\mathrm{full}\;\mathrm{and}\;\mathrm{partial}\;\mathrm{wrapping},\;\mathrm{respectively}\right)$$

(28) $$\lambda_{fv}^{*}=\frac{n_{f}t_{f}E_{f}\varepsilon_{fe}}{f_{c}^{\prime }b}$$

Briefly, the proposed model is summarized by the following steps: (1) the FRP strain efficiency factors (*k_εh_* and *k_εv_*) are determined using Equations (13) and (14), (2) the effective confinement parameters ($\lambda_{fv}^{*}$ and $\lambda_{fh}^{*}$) are calculated by Equations (27) and (28). After obtaining the strength enhancement achieved by the internal steel reinforcement using Equation (25) ($p_{uc}^{u}$/*P_c_*), the enhancement in strength at failure ($p_{cc}^{u}$/*P_c_*) of eccentrically-loaded FRP-confined RC columns can be computed by Equation (26).

## 4. Conclusions

Based on the analysis of 230 results of a total of 155 tested specimens compiled from 19 studies, a new model was provided to estimate the axial compression strength of FRP-confined reinforced rectangular RC columns under eccentric axial loads. The database covers a wide range of test variables such as cross-sectional size, aspect ratio, corner radius, and ratio of internal hoop steel reinforcement, FRP wrapping scheme, and amount of FRP. It was finally shown the proposed model to fit very well with the test results. The main findings of this research are provided as follows:

- Tests on eccentrically-loaded FRP-confined RC columns have shown the increase of the hoop steel reinforcement ratio increases the axial strength capacities. Tests have also shown that the confinement efficiency to decrease as the cross-sectional size and eccentric loading ratio increases.
- The actual rupture strain of the FRP wraps at the corners of the cross sections depends on the ratio of the corner radius to the cross-sectional depth and the eccentricity ratio in addition to the confinement stiffness ratio. Expressions for the actual strain of the FRP hoop at failure of eccentrically loaded RC columns were provided. The model presented in this study is based on limited column tests. Since the FRP hoop rupture strain is more sensitive to column parameters, further experimental and analytical research on tests considering the effects of wider ranges of test parameters to validate the application of the expressions may be necessary.
- For eccentric loads where the longitudinal steel reinforcement starts to yield in tension, the effect of confinement is of secondary importance. In such a case, the effectively confined core concrete area decreases as the eccentricity ratio increases. Therefore, it is now guaranteed by using the proposed model to choose a suitable reinforcement scheme (i.e., longitudinal CFRP strips) and number of CFRP layers for columns that can exhibit an ascending type of response.

## Figures and Tables

**Table 1:** Influence factors of existing model expressions of FRP-confined concrete under eccentric axial compression.

Proposed Model Influence Factor								
FRP Confining Stress	Shape Factor	Corner Radius	FRP Effective Strain	FRP Ratio	Eccentricity Level	Hoop Steel	Slenderness Ratio	Section Size
Currently proposed								
Yes	Yes	Yes	Yes	Yes	Yes	Yes	Yes	Yes
El Maaddawy [29]								
Yes	Yes	No	No	Yes	Yes	No	No	No
Hu et al. [30]								
Yes	Yes	No	No	Yes	Yes	No	Yes	No
Yang et al. [22]								
Yes	Yes	Yes	Yes	Yes	No	No	No	No
Pour et al. [31]								
No	No	Yes	No	No	Yes	No	No	No
Al-Nimry and Neqresh [32]								
Yes	Yes	No	Yes	Yes	No	No	No	No

**Table 2:** Experimental detail of FRP-wrapped unreinforced and RC specimens.

Research Study	Specimens Number, N	Confinement Technique	Wrapping Mode	Width, *b*: mm	Depth, *h*: mm	Height, *H*: mm	Corner Radius, *r_c_*: mm	Cylinder Concrete Strength, *f_c_*	Maximum Load (KN)	Failure Load (KN)
Jaturapitakkul et al. [27]	10	Hoop reinforcement	-	200	300	1200	-	9.9	423.4–791.9	-
Al-Nimry and Neqresh [32]	18	CFRP/hoop reinforcement	Full	200	200	1200	12.5	43.8–52.4	917.6–1903	735.2–1552.3
Darby et al. [33]	5	CFRP	Full	150	150	925–1250	20	28.1–31.5	184–812	-
Shaikh and Alishahi [34]	12	CFRP/hoop reinforcement	Full	175	175	800	20	47	654–1320	403.4–1122.8
Hassan et al. [21]	6	E-Glass/hoop reinforcement	Partial & Full	200	200	1850	13	68–72.1	322–2005	350.3–1959.5
Song et al. [28]	8	CFRP	Full	250	250	1500	25	20	384–1214	322.3–1145.1
Hadi et al. [18]	2	Hoop reinforcement	-	200	200	800	-	73.1	1336–1950	413–467
Allawi et al. [35]	7	CFRP/hoop reinforcement	Full	150	150	700	10	33.2	159–750.8	139.9–622.1
El Maaddawy et al. [29]	9	CFRP/hoop reinforcement	Partial & Full	125	125	1200	10	28.5	92–205	-
Sadeghian et al. [36]	6	CFRP/hoop reinforcement	Full	200	300	2700	15	40	-	156-600
Saljoughian and Mostofinejad [37]	6	CFRP/hoop reinforcement	Partial	133	133	500	8	30	80.7–235.3	61–197.1
El Maaddawy et al. [19]	12	CFRP/hoop reinforcement	Full	110–135	135–160	1300–1400	10	20	105.3–190.2	-
Taranu et al. [38]	4	CFRP/hoop reinforcement	Full	250–300	250–300	1000	35	30	2004.9–2480.7	1988.4–2419.3
Xian et al. [39]	20	CFRP/hoop reinforcement	Partial	250	350	2200	13	17.7–40.5	640-2390	-
Yang et al. [22]	14	CFRP/hoop reinforcement	Partial & Full	150	200	1200	15	46.7	401.5–1004.9	340.9–854.3
Elsayed et al. [40]	3	CFRP/hoop reinforcement	Partial & Full	100	150	1700	20	20.6	258–296	224.3–248.9
Zhou and Huang [41]	7	CFRP/hoop reinforcement	Partial & Full	150	200	1200	20	33.2	328.5–878	-
Jinglong et al. [42]	3	CFRP/hoop reinforcement	Full	120	150	950	15	26.6–27.7	163–621	-
Lin et al. [43]	3	CFRP/hoop reinforcement	Full	290	435	1300	35	45	2693.9–5123.2	-

**Table 3:** Mechanical properties of steel reinforcement and FRP material.

Source	Longitudinal Steel *f_yl_* (MPa)	Hoop Steel *f_yh_* (MPa)	FRP Material			
			*E_f_* (GPa)	*f_f_* (MPa)	*t_f_* (mm)	*ε_fu_* (%)
Jaturapitakkul et al. [27]	282.8 (Φ12)	283.2 (Φ6)	-	-	-	-
Al-Nimry and Neqresh [32]	420 (Φ10)	570 (Φ6)	240	3800	0.170	1.55
Darby et al. [33]	550 (Φ12)	-	214	3103	0.160	1.45
Shaikh and Alishahi [34]	500 (Φ12)	500 (Φ6)	230	3450	0.130	1.50
Hassan et al. [21]	400 (Φ16)	240 (Φ8)	70	2250	0.170	2.80
Song et al. [28]	337.6 (Φ14)	421.9 (Φ6)	222	3500	0.167	1.70
Hadi et al. [18]	564 (Φ12)	516 (Φ8)	-	-	-	-
Allawi et al. [35]	566-652 (Φ12)	566 (Φ6)	230	3450	0.130	1.50
El Maaddawy et al. [29]	550 (Φ10)	550 (Φ6)	230	3450	0.130	1.50
Sadeghian et al. [36]	465 (Φ12)	325 (Φ6.5)	242	3860	0.250	1.60
Saljoughian and Mostofinejad [37]	406 (Φ10)	550 (Φ8)	230	3900	0.170	1.50
El Maaddawy et al. [19]	520 (Φ10)	300 (Φ6)	230	3450	0.130	1.50
Yang et al. [22]	403.7 (Φ14)	365.3 (Φ6)	240	4250	0.167	1.77
Taranu et al. [38]	300 (Φ12)	210 (Φ6)	234.5	3793	0.340	1.50
Xian et al. [39]	365 (Φ18)	365 (Φ8)	230	4700	0.111	2.00
Elsayed et al. [40]	420 (Φ12)	290 (Φ8)	240	3800	0.170	1.55
Zhou and Huang [41]	365.9 (Φ14)	361.6 (Φ6)	87.7	2800	0.169	3.20
Jinglong et al. [42]	445 (Φ10)	445 (Φ4)	230	4700	0.111	2.00
Lin et al. [43]	491 (Φ20)	380 (Φ8)	238.8	3993.3	0.334	1.68

**Table 4:** Regression parameters for the proposed expressions.

Equation (13): Strain Efficiency Factor of FRP in Hoop Axis						
*B*_1_	*B*_2_	*B*_3_	*B*_4_	*B*_5_	*B*_6_	*B*_7_
0.626	0.454	0.900	−0.120	0.220	0.06	-
Equation (14): Strain efficiency factor of FRP in longitudinal axis						
*B*_1_	*B*_2_	*B*_3_	*B*_4_	*B*_5_	*B*_6_	*B*_7_
0.205	10.2660	−9.097	0.678	-	-	-
Equation (15): Peak load ratio of unwrapped RC columns (0.1 ≤ e/h ≤ 0.33)						
*B*_1_	*B*_2_	*B*_3_	*B*_4_	*B*_5_	*B*_6_	*B*_7_
2.601	0.379	12.086	3.060	−2.348	0.014	0.268
Equation (15): Peak load ratio of unwrapped RC columns (0.4 ≤ e/h ≤ 1)						
*B*_1_	*B*_2_	*B*_3_	*B*_4_	*B*_5_	*B*_6_	*B*_7_
−0.390	−0.524	2.367	2.801	−0.956	0.009	0.292
Equation (22): Peak load ratio of FRP-wrapped RC columns						
*B*_1_	*B*_2_	*B*_3_	*B*_4_	*B*_5_	*B*_6_	*B*_7_
2.120	0.719	3.304	0.613	0.377	−3.333	3.238 × 10^−3^
Equation (25): Failure load ratio of unwrapped RC columns						
*B*_1_	*B*_2_	*B*_3_	*B*_4_	*B*_5_	*B*_6_	*B*_7_
1.683	0.225	−1.563	1.212	-	-	-
Equation (26): Failure load ratio of FRP-wrapped RC columns						
*B*_1_	*B*_2_	*B*_3_	*B*_4_	*B*_5_	*B*_6_	*B*_7_
0.304	0.355	−1.296	6.716	0.771	−1.670	-

**Table 5:** Model predictions of FRP strain efficiency factor (*k_εh_*, *k_εv_*) of FRP-confined concrete.

Model	Prediction of *k_εh_*				Prediction of *k_εv_*			
	Averaged (Model/Test) (%)	Average Absolute Error (%)	Mean (%)	Standard Deviation (%)	Averaged (Model/Test) (%)	Average Absolute Error (%)	Mean (%)	Standard Deviation (%)
Currently proposed	99.95	17.20	4.67	22.42	100	3.34	0.19	4.83
Barrington et al. [54]	94.27	79.18	108.60	110.90	-	-	-	-
Wang et al. [11]	215.06	264.12	1318.48	303.77	-	-	-	-
Hadi et al. [51]	189.10	195.49	739.90	226.07	-	-	-	-
Ozbakkaloglu [60]	250.56	307.13	1756.29	337.40	-	-	-	-
Song et al. [28]	254.77	304.86	1660.88	320.99	-	-	-	-
Hany et al. [61]	231.61	268.75	1323.07	291.81	-	-	-	-
Wang et al. [62]	207.44	252.52	1242.84	298.45				
ACI 440.2R [63]	212.31	239.21	1071.48	267.50	-	-	-	-
Yang et al. [22]	-	-	-	-	141.34	69.71	60.38	56.55

**Table 6:** Comparison between reduction in tested and predicted peak strengths for selected unconfined RC columns tested under different eccentric loading levels.

Specimen	Tested Peak Load (KN)	Reduction in Strength (Test) (%)	*e/h*	Proposed *γ*	Reduction in Strength (Model) (%)
Jinglong et al. [42]					
Z-10	496	-	0.133	0.742	-
Z11	163	−67.14	0.600	0.261	−64.86
Darby et al. [33]					
SE2u	774	-	0.100	0.799	-
SE3u	395	−48.97	0.327	0.481	−39.83
SE4u	184	−76.23	0.720	0.199	−75.08
Saljoughian and Mostofinejad [37]					
U-30	352	-	0.226	0.603	-
U-60	197	−44.12	0.451	0.364	−39.68
U-90	119	−66.31	0.677	0.219	−63.61
U-120	81	−77.07	0.902	0.132	−78.05
El Maaddawy et al. [19]					
SN-e1	150	-	0.460	0.357	-
SN-e2	105	−29.89	0.600	0.261	−26.93
R2N-e1	149	-	0.460	0.357	-
R2N-e2	111	−25.57	0.600	0.261	−26.93
Yang et al. [22]					
SR	733	-	0.250	0.571	-
LR	402	−45.19	0.500	0.326	−42.89

**Table 7:** Comparison between reduction in tested and predicted peak strengths for selected FRP-confined RC columns tested under different eccentric loading levels.

Specimen	Tested Peak Load (KN)	Reduction in Strength (Test) (%)	*e/h*	Proposed *γ*	Reduction in Strength (Model) (%)
Jinglong et al. [42]					
Z-3	621	-	0.133	0.659	-
Z-8	195	−68.59	0.600	0.209	−68.30
El Maaddawy [29]					
FW-e1	295	-	0.300	0.417	-
FW-e2	205	−30.51	0.432	0.302	−27.55
FW-e3	157	−46.78	0.568	0.223	−46.45
FW-e4	95	−67.79	0.860	0.126	−69.68
PW-e1	275	-	0.300	0.417	-
PW-e2	200	−27.27	0.432	0.302	−27.55
PW-e3	150	−45.45	0.568	0.223	−46.45
PW-e4	93	−66.18	0.860	0.126	−69.68
Sadeghian et al. [36]					
S200-L2T	491	-	0.667	0.182	-
S300-L2T	284	−42.16	1	0.099	−45.53
S200-L4T	600	-	0.667	0.182	-
S300-L4T	356	−40.67	1	0.099	−45.53
El Maaddawy et al. [19]					
SF-e1	186	-	0.460	0.283	-
SF-e2	134	−27.60	0.600	0.209	−26.29
Darby et al. [33]					
SE2	812	-	0.100	0.728	-
SE3	464	−42.86	0.327	0.389	−46.43
SE4	249	−69.33	0.720	0.164	−77.45
Hadi et al. [18]					
1HC25	2076	-	0.125	0.675	-
1HC50	1433	−30.97	0.250	0.475	−29.60
3HC25	2269	-	0.125	0.675	-
3HC50	1534	−32.39	0.250	0.475	−29.60
Hassan et al. [21]					
US-G1F	1640	-	0.125	0.675	-
UL-G1F	455	−72.26	0.625	0.198	−70.64
US-G2F	2005	-	0.125	0.675	-
UL-G2F	492	−75.46	0.625	0.198	−70.63
Elsayed et al. [40]					
C02	375	-	0.167	0.598	-
C06	258	−31.20	0.333	0.383	−35.91
C03	434	-	0.167	0.598	-
C07	296	−31.79	0.333	0.383	−35.91
Shaikh and Alishahi [34]					
CR11	1229	-	0.143	0.641	-
CR12	1098	−10.66	0.200	0.545	−15
CR13	798	−35.07	0.286	0.433	−32.46
CR21	1320	-	0.143	0.641	-
CR22	1020	−22.72	0.200	0.545	−15
CR23	884	−33.03	0.286	0.433	−32.46
CR31	1283	-	0.143	0.641	-
CR32	998	−22.21	0.200	0.545	−15
CR33	840	−34.53	0.286	0.433	−32.46

**Table 8:** Comparison between tested and predicted failure strength ratios of unwrapped RC columns tested under different eccentric loading levels.

Reference	Specimen Code	*P_uc_^u^/P_cc_* (Test)	*P_uc_^u^/P_cc_* (Model)
Saljoughian and Mostofinejad [37]	U-60	0.86	0.83
	U-90	0.83	0.77
	U-120	0.76	0.69
Yang et al. [22]	SR	0.85	0.89
	LR	0.85	0.84
Allawi et al. [35]	12C45	0.84	0.84
	12C90	0.60	0.74
	6C90	0.86	0.82
Song et al. [28]	SSR-3-0	0.84	0.84
	SSR-4-0	0.84	0.77
	SSR-1-0	0.85	0.84
	SSR-2-0	0.84	0.87
Al-Nimry and Neqresh [32]	C35-U-A	0.82	0.88
	C35-U-B	0.90	0.88
	C50-U-A	0.90	0.87
	C50-U-B	0.97	0.87
	C50-U-C	0.86	0.88
	C65-U-A	0.82	0.86
	C65-U-B	0.80	0.87
Average		0.84	0.83

**Table 9:** Comparison between experimental results of selected RC columns with FRP wraps or a combination of FRP hoop and longitudinal reinforcement.

Specimen Code	Eccentricity Ratio (e/h)	Wrapping System	Peak Load (KN)	Failure Load (KN)	Strength Enhancement at Peak (%)	Strength Enhancement at Failure (%)
Hassan et al. [21]						
US-G1F	0.1250	FRP hoop	1640	1532	-	-
US-G2F	0.1250	FRP hoop	2005	1959	22.3	27.9
UL-G1F	0.6250	Hoop and longitudinal FRP	455	350	-	-
UL-G2F	0.6250	Hoop and longitudinal FRP	492	439	8.1	25.2
Al-Nimry and Neqresh [32]						
C50-C	0.2500	FRP hoop	1469	1417	-	-
C65-C	0.3250	FRP hoop	1190	1033	−19	−27.1
C50-LC	0.2500	Hoop and longitudinal FRP	1430	1325	-	-
C65-LC	0.3250	Hoop and longitudinal FRP	1239	1166	−13.4	−11.99

## Data Availability

The data presented in this study are available on request from the corresponding author.

## Abbreviations

The symbols used in this paper are as follows:

b*&*h	width and depth of rectangular cross section, respectively (mm)
D	equivalent diameter of rectangular section (mm)
$r_{c}$	corner radius (mm)
$A_{g}$	area of rectangular cross section with rounded corners (mm^2^)
$H_{c}$	clear height of column between end corbels
$n_{f}$	total number of layers of full FRP wraps
$N_{s}$	total number of layers of partial FRP wraps
$t_{f}$	thickness of one FRP layer (mm)
$w_{f}$	width of FRP strip (mm)
$s_{f}$	center-to-center spacing of FRP strips (mm)
$E_{f}$	elastic modulus of elasticity of FRP (MPa)
$f_{f}$	tensile strength of FRP (MPa)
$\rho_{f}$	volumetric ratio of FRP wraps
$k_{ef}$	coefficient for effectiveness of FRP wraps
$k_{es}$	coefficient for effectiveness of internal steel ties
$k_{\varepsilon h}$	strain efficiency factor of lateral FRP wraps
$k_{\varepsilon v}$	strain efficiency factor of longitudinal FRP
$\varepsilon_{fe}$	effective tensile strain of FRP at rupture
$\varepsilon_{fu}$	ultimate tensile strain of FRP at rupture
$f_{yh}$	yield strength of hoop steel bars (MPa)
$f_{yl}$	yield strength of longitudinal steel bars (MPa)
$f_{c}^{\prime }$	strength of unconfined concrete 150 mm × 300 mm cylinders (MPa)
$f_{cd}^{\prime }$	strength of unconfined concrete 100 mm × 200 mm cylinders (MPa)
$f_{cu}^{\prime }$	strength of unconfined concrete 150 mm × 150 mm cubes (MPa)
$\varepsilon_{co}$	unconfined concrete strain (mm/mm)
$k_{sf}$	shape factor to account for a rectangular cross section with rounded corners
AAE	average absolute error
MSE	mean square error
SD	standard deviation
$R_{sh}$	proposed confinement stiffness ratio of FRP in hoop axis
$R_{sv}$	proposed confinement stiffness ratio of FRP in vertical axis
$c_{x}$*&*$c_{y}$	concrete core dimensions to the center line of the hoops along *x* and *y* directions (mm)
$\rho_{cc}$	ratio of longitudinal reinforcement area divided by concrete core area
$A_{shx}$*&*$A_{shy}$	cross-sectional areas of hoop reinforcement along *x* and *y* directions (mm^2^)
$\rho_{sx}$*&*$\rho_{sy}$	volumetric ratios of hoop reinforcement in *x* and *y* directions
$\rho_{eh}$	effective volumetric ratio of steel ties
$w_{xi}$*&*$w_{yi}$	clear distances between longitudinal bars along *x* and *y* directions (mm)
$s^{\prime }$	vertical clear spacing between steel ties (mm)
s	center-to-center spacing between steel ties (mm)
$\rho_{s}$	ratio of longitudinal steel reinforcement
$A_{s}$	total area of longitudinal steel reinforcement (mm^2^)
n	total number of tested specimens
α	coefficient to account for the unconfined concrete strength effect
γ	coefficient to account for the effect of increased eccentricity ratio
$N_{uo}$	axial loading capacity of columns (KN)
$P_{uc}$	peak axial load of unwrapped RC columns (KN)
$P_{cc}$	peak axial load of FRP-wrapped columns (KN)
$P_{uc}^{u}$	failure axial load of unwrapped columns (KN)
$P_{cc}^{u}$	failure axial load of FRP-wrapped columns (KN)
$P_{c}$	axial load of concrete cylinder (KN)
$k_{se}$	proposed coefficient to account for the effect of FRP wrapping schemes
